# Inducibility of Multiple Ventricular Tachycardia’s during a Successful Ablation Procedure Is a Marker of Ventricular Tachycardia Recurrence

**DOI:** 10.3390/jcm12113660

**Published:** 2023-05-25

**Authors:** Johnatan Nissan, Avi Sabbag, Roy Beinart, Eyal Nof

**Affiliations:** 1 Department of Diagnostic Imaging, Sheba Medical Center, Ramat Gan 52621, Israel; johnatan.n@gmail.com; 2 Sackler School of Medicine, Tel Aviv University, Tel Aviv 69978, Israel; avisabbag@gmail.com (A.S.); roy.beinart@sheba.health.gov.il (R.B.); 3 Davidai Arrhythmia Center, Leviev Heart Center, Sheba Medical Center, Ramat Gan 52621, Israel

**Keywords:** ventricular tachycardia, ablation, arrhythmia recurrence

## Abstract

Even after a successful ventricular tachycardia ablation (VTA), some patients have recurrent ventricular tachycardia (VT) during their follow-up. We assessed the long-term predictors of recurrent VT after having a successful VTA. The patients who underwent a successful VTA (defined as the non-inducibility of any VT at the procedure’s end) in 2014–2021 at our center in Israel were retrospectively analyzed. A total of 111 successful VTAs were evaluated. Out of them, 31 (27.9%) had a recurrent event of VT after the procedure during a median follow-up time of 264 days. The mean left ventricular ejection fraction (LVEF) was significantly lower among patients with recurrent VT events (28.9 ± 12.67 vs. 23.53 ± 12.224, *p* = 0.048). A high number of induced VTs (>two) during the procedure was found to be a significant predictor of VT recurrence (24.69% vs. 56.67%, 20 vs. 17, *p* = 0.002). In a multivariate analysis, a lower LVEF (HR, 0.964; *p* = 0.037) and a high number of induced VTs (HR, 2.15; *p* = 0.039) were independent predictors of arrhythmia recurrence. The inducibility of more than two VTs during a VTA procedure remains a predictor of VT recurrence even after a successful VT ablation. This group of patients remains at high risk for VT and should be followed up with and treated more vigorously.

## 1. Introduction

Ventricular tachycardia ablation (VTA) is a preventive treatment of recurrent ventricular tachycardia (VT) and may reduce mortality among subjects with structural heart disease [1,2]. While the primary endpoint of VTA may be a subject of debate, it is agreed that a minimal endpoint of the non-inducibility of clinical VT is desirable. However, many aim to reach the endpoint of the non-inducibility of any VT. In spite of the above, many patients still experience VT after an ablation. There are several known long-term outcomes predictors, such as VT inducibility at the end of the procedure, being of an older age, having a severely reduced left ventricular ejection fraction (LVEF), and the failure of late potential abolition [1,3,4,5].

However, there are scarce data on the long-term recurrence of VT after what is deemed a successful VTA. This study aimed to assess the possible predictors of VT recurrence even after achieving the desired endpoint of non-inducibility at the end of the ablation procedure.

## 2. Material and Methods

### 2.1. Study Design

This study was approved by the Institutional Review Board of Sheba Medical Center, Tel Hashomer. After retrospectively reviewing a total of 193 procedures, we identified 108 patients who underwent 111 (57.5%) catheter successful ablations of VT between 2014 and 2021 at Davidai Arrhythmia Center at Sheba Medical Center in Israel. Of the other 82 procedures, 19 failed, 27 had partial success, and the inducibility was not checked in the remaining procedures. The data were collected and analyzed in 2021–2022.

#### 2.1.1. Ablation Procedure

Electro-anatomical mapping (EAM) was performed (Carto 3, Biosense Webster, Diamond Bar, CA, USA) using an open irrigated catheter with a 3.5 mm tip (THERMOCOOL SMARTTOUCH™ SF Catheter, Biosense Webster, Diamond Bar, CA, USA). Voltage maps were created during sinus rhythm. Peak-to-peak bipolar electrogram amplitude <0.5 mV was defined as a dense scar, and a voltage of ≥0.5 and <1.5 mV was defined as a scar border zone. Epicardial mapping and ablation were carried out when necessary. EAM was performed with the Pentaray™ Catheter (Biosense Webster, Diamond Bar, CA, USA). Most of the endocardial mapping was performed with a retrograde approach, except 5 cases for which a trans-septal approach was used. In cases in which a epicardial approach was used, the epicardial map was also performed with the Pentaray catheter.

All inducible sustained monomorphic ventricular tachycardias (SMVTs) were targeted for ablation. If VT was mappable, sites were targeted for ablation if pacing entrained the SMVT with concealed fusion and a post-pacing interval (PPI) within 30 ms of the VT cycle length (CL) or by activation mapping. In any case, scar substrate modification was performed. Sites with low-amplitude fractionated electrograms that had long stimulation to QRS, late potentials, or the best-pace map sites were targeted. Pace mapping and entrainment mapping utilized unipolar stimuli with a strength of 10 mA and pulse width of 2 ms Radiofrequency (RF) energy was delivered at a power of 35 to 50 Watts, targeting an impedance drop of at least 10 ohms. The endpoint of all procedures was the non-inducibility of any VT with programmed stimulation (PS) at a basic drive train of 600 and 400 ms with up to 3 extrastimuli. The procedures were performed at a targeted ACT of >300 s. A procedure was defined as successful if the patient was non-inducible for any VT following PS. Partial success was declared when only the clinical VT was no longer inducible, and acute procedural failure was declared if the clinical VT was still inducible.

#### 2.1.2. Study Population

The study’s inclusion criteria were patients who underwent VTAs in our institution due to VT events and had a successful VTA procedure. Only patients with inducibility of at least one VT during the procedure were included. All patients were followed up with at our institution, and device interrogation was performed regularly during the follow-up visits (the day after as well as one month, three months, and every six months after the index procedure). Recurrent VT during follow-up was defined as any event of sustained VT (even if self-terminated and tolerable) documented by the patient’s implantable cardioverter-defibrillator (ICD) device or in the patient’s medical chart. Patients were divided into two groups. Those who had recurrent VT events during follow-up and those who did not. In patients who underwent an additional VTA after the index VTA, the follow-up period evaluated in the study was only the time between the two VTAs.

Each group’s main baseline and ablation characteristics were extracted manually from the patient’s medical documents. A baseline characteristic was noted if it was defined by the treating physician and was documented in at least two medical documents. Permanent atrial fibrillation (AF) was defined by the 2020 European Society of Cardiology Guidelines for diagnosing and managing atrial fibrillation [6]. Laboratory information was only noted if collected at least 24 h before the procedure, and the closest results to the procedure were assessed. The patients were divided into tertiles by the number of induced VTs during the procedure. The subjects at the highest tertile were categorized as having a “high” number of induced VTs, and the lower two tertiles were categorized as having a “low” number of induced VTs (calculated as a cut-off value of ≥3).

Major complication was defined as one of the following complications: having a cardiac arrest during the operation, post-operative stroke, and pericardial tamponade. A minor complication was defined as a significant groin hematoma (which required treatment with at least one blood unit) or the need for femoral artery intervention.

### 2.2. Statistical Analysis

The univariate analyses included in this study were the Chi-square test or Fisher’s test for categorical variables and the student’s *t*-test for continuous variables. Variables with a calculated *p*-value ≤ 0.05 in the univariate analyses were included in the multivariate analysis. The multivariate analysis used in the study was Cox regression. The univariate survival analyses used in this study were the Kaplan–Meier curve and the log-rank test. SPSS (version 24.0, IBM, Armonk, NY, USA), R software package (version 4.2.2), and RStudio (version 2023.03.1+446, R Studio PBC, Boston, MA, USA) were used for the statistical analyses in this study.

## 3. Results

The study included 111 procedures in a total of 108 patients, 30 (27%) of whom had a recurrent VT event after a total median follow-up time of 264 days (IQR, 56–719 days) after the index procedure. A total of 50/111 (45%) had a follow-up time of more than 1 year (either they died or were lost in the follow-up). The median VT-free survival time of the recurrent VT group was 100 days (IQR, 32–529 days), and the median VT-free survival time of the entire cohort was 183 days (IQR, 36–583 days). The 1-year VT recurrence rate of the cohort was 18/50 (36%) among the patients who survived the first year after the procedure and had available data. Out of the 30 patients in the VT recurrence group, 12 (40%) were treated with shock, 14 (46.67%) only with ATP, and 4 (13.33%) were not treated with either shock or ATP.

During the procedure, the mean subject’s age was 66.08 ± 9.71 years, and most of the subjects were males (92.8%). Seventy-one (64%) patients had hypertension at baseline, 28 (25.2%) had diabetes mellitus, and 22 (19.8%) had chronic kidney disease. Thirty-seven subjects (33.3%) had a background of AF, 10 had permanent AF, and 27 had non-permanent AF (24.3% and 9%, respectively). Ninety-five (85.6%) subjects had heart failure, without a significant difference between the study’s groups (84% vs. 90% for those with non-recurrent VT vs. recurrent VT, respectively). The LVEF was significantly lower in the group of patients who had a VT event after the procedure, compared with that of the group that did not have a VT event (23.53 ± 12.224 vs. 28.9 ± 12.67, respectively, *p* = 0.048). Twenty-eight patients (25.2%) had a history of a previous VTA, without a significant difference between the two groups (25.9% vs. 23.3%; *p* = 0.781). Most patients had ischemic VT etiology (88, 79.3%) and were treated chronically with beta-blockers before the procedure (96, 86.5%) (Table 1).

**Figure 1:**
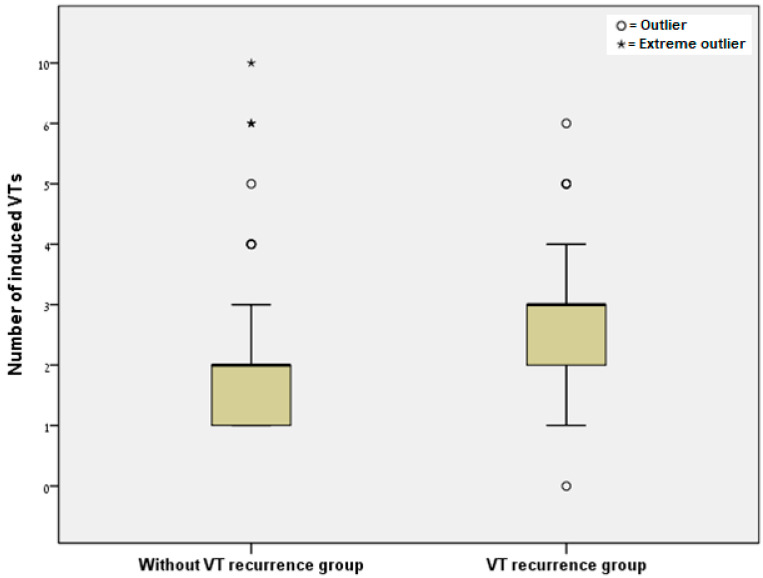
Box plot of the number of induced VTs in the group who did not have VT recurrence vs. the group with VT recurrence (*p* = 0.088). Outlier—3rd quartile + 1.5 × interquartile range or 1st quartile–1.5 × interquartile range; Extreme outlier—3rd quartile + 3 × interquartile range or 1st quartile–3 × interquartile range.

Thirty-seven (33.33%) procedures were performed with the assistance of an ECMO device, 71 (63.96%) patients were ablated under general anesthesia, and 54 (48.65%) required catecholamines during and after the procedure. The mean number of induced VTs per procedure was 2.27 ± 1.49. The group without VT recurrence had a lower mean of induced VTs than that of the group with VT recurrence (2.12 ± 1.49 vs. 2.67 ± 1.42, respectively, *p* = 0.088; Figure 1).

**Figure 2:**
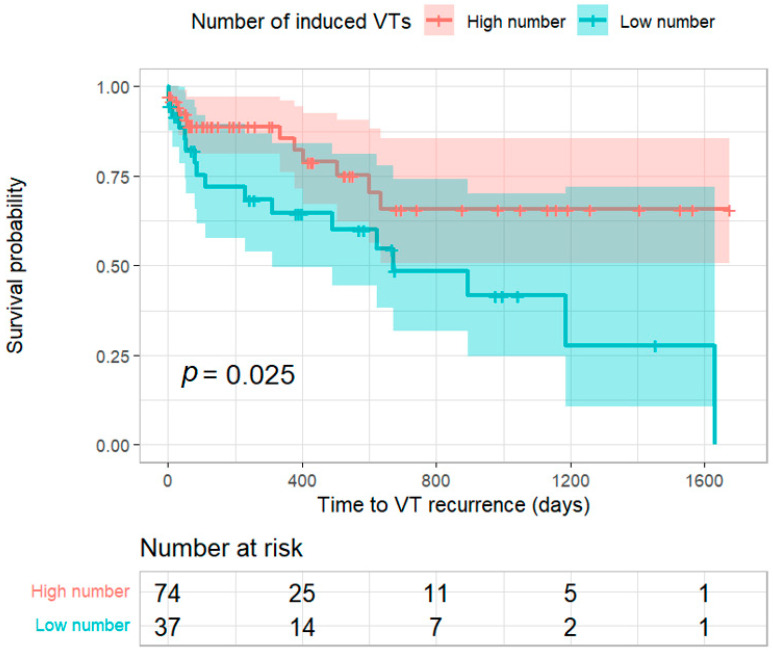
Kaplan–Meier survival curve for a high number of induced VTs during the ablation (*p*-value = 0.025).

A high number of induced VTs (>2) was significantly less common in the group which did not have VT recurrence (24.69% vs. 56.67%, 20 vs. 17, respectively, *p* = 0.002). The log-rank test for a high number of induced VTs also indicated this significant association (*p* = 0.025; Figure 2). Forty-seven (42.34%) patients had a posterior-lateral scar, 35 (31.53%) had an antero-septal scar, 17 (15.32%) had a posterior-right scar, 2 (1.8%) had an epicardial scar, and 10 (9.01%) patients did not have a scar (Table 2). The location of the scar did not predict recurrence.

A total of 85 (76.6%) patients were on anti-arrhythmic medication prior to the procedure. Anti-arrhythmic medications included Amiodarone (64 (57.7%)), Sotalol (19 (17.1%)), and Mexiletine (19 (17.1%)). Out of this patient group, 94 (84.7%) were discharged on the same anti-arrhythmic medication, and 17 (15.3%) were discharged on another anti-arrhythmic medication. This did not differ between groups (10 (12.3%) vs. 7 (23.3%); *p* = 0.233).

Post-ablation major complications were observed in two (1.8%) patients and minor complications in two (1.8%) patients. The prevalence of any type of complication did not differ between groups. Seventeen (15.32%) patients died during the follow-up time: nine (11.11%) patients in the group that did not have VT recurrence and eight (26.67%) patients in the VT recurrence group (*p* = 0.071). The mean mortality time after ablation was 421.94 ± 554.92 days (172.44 ± 361.93 days in the group that did not have VT recurrence, and 702.62 ± 619.75 days in the VT recurrence group (*p* = 0.045)) (Table 3).

In the multivariate Cox regression analysis, the LVEF (Hazard ratio [HR], 0.964; 95% confidence interval [95% CI], 0.932–0.998; *p* = 0.037) and a high number of induced VTs (HR, 2.15; 95% CI, 1.04–4.45; *p* = 0.039) were significant predictors of VT recurrence (Table 4).

## 4. Discussion

To the best of our knowledge, this is the first study to separately analyze the predictors of VT recurrence after what is acutely considered a successful VTA. Although several predictors for VT recurrence have been described previously in the literature, these studies did not characterize this specific sub-group of patients, which is typically considered a lower-risk group for VT recurrence and mortality [3,7]. Our study indicates two possible predictors for VT recurrence after a successful VTA—a lower LVEF and a high number of induced VTs during the procedure.

Although a lower LVEF [8,9,10,11,12,13] and a high number of induced VTs during ablation [9,12,14] have been previously described as predictors of VT recurrence after VTA, our data are unique in the fact that these variables remain as predictors even after a successful VTA. This finding is surprising as, at the end of the procedure, all our patients by definition were non-inducible for any VT. Thus, the substrate creating the inducible VT seen at the beginning of the procedure was considered as successfully targeted. In spite of the above, these patients had a higher VT recurrence rate, most probably reflecting the presence of a more complex substrate.

The predictors demonstrated in our study can serve as a possible indicator for the unknown underlying mechanism through which VTs are developed after a successful VTA. As a lower LVEF has already been assessed as a predictor of VTA in the study of Haanschoten et al. [8], it was hypothesized that the underlying mechanism might be related to a possible more extensive and complex arrhythmogenic substrate in patients with a lower LVEF, which can disrupt the elimination of all the possible arrhythmogenic pathways during the procedure. Furthermore, de Riva et al. [9] suggested that not all VTs have a fixed reentry mechanism, especially among patients with enhanced cardiac remodeling and heart failure, which might generate additional focal reentry cycles that are not accurately reproducible by programmed electric stimulation. In our study, only those with non-inducible VTAs at the conclusion of the procedure were included, indicating that all the “fixed” arrhythmogenic pathways were probably not inducible at the end of the ablation. This might strengthen the hypothesis of focal arrhythmic mechanisms that generate VT recurrence, foci that electric stimulation could not generate during the procedure. The same hypothesis can also be implicated by the second predictor found in the study—a high number of induced VTs. A higher number of induced VTs may indicate a more complex scar substrate tissue which may prevent the stimulation of these focal or even reentrant pathways.

An alternative explanation could be a deeper (intramural) substrate that was not mapped or recognized during the procedure. Previous studies [15,16] have found that intramural substrates account for a relatively large number of VTs, even in ischemic cardiomyopathy patients. This intramural substrate could be identified mainly during VT by electro-anatomical mapping. If the VT arising from such a substrate is non-inducible during the procedure, it might be challenging to identify the substrate and target it.

The main clinical implication of our study is that patients with more than two inducible VTs during a VTA procedure should be followed up rigorously and anti-arrhythmic medication should most probably be continued even if it is a successful procedure. Further studies should assess the possible underlying mechanism of VT recurrence among patients who underwent a successful VTA to evaluate the causes of VT recurrence after a VTA.

The current analysis indicates a 36% one-year VT recurrence rate after a successful VTA. Comparisons to previous studies’ outcomes are relatively limited due to the lack of standardization in the outcome time. Breitenstein et al. [17] recently assessed the one-year recurrence rate of VT after a successful VTA as 45% in a large cohort of patients with structural heart diseases, and Okubo et al. [18] evaluated it was 16% among patients with a late potential abolition and 32% without one. Other studies with relatively small non-inducibility study groups evaluated this recurrence rate as 0–40% among patients with various clinical characteristics [9,11,19,20]. The high recurrence rate heterogeneity might be related to the lack of study population uniformity and different patient baseline characteristics.

Our study’s main limitation is the demonstration of a causal link only between successful VTA characteristics and VT recurrence without investigating the underlying reason for this association. Further studies should assess the mechanism of this association. Another limitation is the study’s retrospective design which might include natural retrospective biases, such as information and selection biases, and the relatively low number of patients, which might fail to recognize other predictors.

## 5. Conclusions

The inducibility of many VTs during a VTA procedure predicts VT events in the long term, even if the patient is deemed non-inducible for any VT at the termination of the procedure.

## Figures and Tables

**Table 1:** Baseline characteristics: non-VT recurrence group vs. VT recurrence group.

	All (*n* = 111)	Non-VT Recurrence Group (*n* = 81)	VT Recurrence Group (*n* = 30)	*p*-Value
Age (years)	66.08 ± 9.71	66.57 ± 9.61	64.77 ± 10	NS
Gender (male)	103 (92.8%)	76 (93.8%)	27 (90%)	NS
Hypertension	71 (64%)	53 (65.4%)	18 (60%)	NS
Diabetes mellitus	28 (25.2%)	20 (24.7%)	8 (26.7%)	NS
Heart failure	95 (85.6%)	68 (84%)	27 (90%)	NS
- LVEF	27.45 ± 12.73	28.9 ± 12.67	23.53 ± 12.22	0.048
- LVEF < 30	60 (54.1%)	40 (49.4%)	20 (66.7%)	0.102
- LVEF of patients with ischemic etiology	25.47 ± 11.11	26.69 ± 11.56	22.21 ± 9.26	0.092
- LVEF of patients with non-ischemic etiology	35 ± 15.67	37.24 ± 13.55	28.83 ± 20.74	NS
Chronic kidney disease	22 (19.8%)	16 (19.8%)	6 (20%)	NS
Atrial fibrillation	37 (33.3%)	27 (33.3%)	10 (33.3%)	NS
- Non-Permanent	27 (24.3%)	18 (22.2%)	9 (30%)	NS
- Permanent	10 (9%)	9 (11.1%)	1 (3.3%)	0.282
Past VTA	28 (25.2%)	21 (25.9%)	7 (23.3%)	NS
Prior ICD	96 (86.5%)	67 (82.7%)	29 (96.7%)	0.065
Ischemic VT etiology	88 (79.3%)	64 (79%)	24 (80%)	NS
Chronic use of beta-blockers	96 (86.5%)	73 (90.1%)	23 (76.7%)	0.2
Hemoglobin (g/dL)	12.38 ± 1.89	12.45 ± 1.99	12.21 ± 1.65	NS
Platelets count (×10^9^/L)	197.72 ± 66.76	196.89 ± 69.34	199.63 ± 61.65	NS
CRP (mg/L)	39.02 ± 61.86	41.36 ± 64.47	32.35 ± 55.4	NS
Creatinine (mg/dL)	1.23 ± 0.92	1.2 ± 1.04	1.28 ± 0.6	NS

Mean ± standard deviation; VT = ventricular tachycardia; LVEF = left ventricular ejection fraction; VTA = ventricular tachycardia ablation; ICD = implantable cardioverter-defibrillator; CRP = C-reactive protein; NS = not significant, *p*-value > 0.3.

**Table 2:** Ablation characteristics: non-VT recurrence group vs. VT recurrence group.

	All (*n* = 111)	Non-VT Recurrence Group (*n* = 81)	VT Recurrence Group (*n* = 30)	*p*-Value
Ablation with ECMO	37 (33.33%)	30 (37.04%)	7 (23.33%)	0.174
General anesthesia	71 (63.96%)	51 (62.96%)	20 (66.66%)	NS
Amines use within 24 h	54 (48.65%)	38 (46.91%)	16 (53.33%)	NS
High number of induced VTs (>2)	37 (33.33%)	20 (24.69%)	17 (56.67%)	0.002
Number of induced VTs	2.27 ± 1.49	2.12 ± 1.49	2.67 ± 1.42	0.088
Scar location				
- Antero-septal	35 (31.53%)	26 (32.1%)	9 (30%)	NS
- Posterior-lateral	47 (42.34%)	33 (40.74%)	14 (46.67%)	NS
- Posterior-right	17 (15.32%)	13 (16.05%)	4 (13.33%)	NS
- Epicardial	2 (1.8%)	0 (0%)	2 (6.67%)	NS
- Without scar	10 (9.01%)	9 (11.11%)	1 (3.33%)	NS

Mean ± standard deviation; VT = ventricular tachycardia; ECMO = extracorporeal membrane oxygenation; NS = not significant, *p*-value > 0.3.

**Table 3:** Ablation outcomes: non-VT recurrence group vs. VT recurrence group.

	All (*n* = 111)	Non-VT Recurrence Group (*n* = 81)	VT Recurrence Group (*n* = 30)	*p*-Value
Major complications	2 (1.8%)	2 (2.47%)	0 (0%)	NS
Minor complications	2 (1.8%)	1 (1.23%)	1 (3.33%)	NS
Mortality	17 (15.32%)	9 (11.11%)	8 (26.67%)	0.071
- Time to mortality (days)	421.94 ± 554.92	172.44 ± 361.93	702.62 ± 619.75	0.045

Mean ± standard deviation; VT = ventricular tachycardia; NS = not significant, *p*-value > 0.3.

**Table 4:** Multivariate Cox regression of the risk for VT recurrence.

	HR (95% CI)	*p*-Value
LVEF *	0.964 (0.93–1)	0.037
High vs. low number of induced VTs	2.15 (1.04–4.45)	0.039

* LVEF as a continuous variable; VT = ventricular tachycardia; HR = hazard ratio; CI = confidence interval; LVEF = left ventricular ejection fraction.

## Data Availability

Anonymized data are available on request from the corresponding author.
